# Prevalence of anxiety, depression and post-traumatic stress disorder among Ebola survivors in northern Sierra Leone: a cross-sectional study

**DOI:** 10.1186/s12889-020-09507-6

**Published:** 2020-09-11

**Authors:** Abdulai Jawo Bah, Peter Bai James, Nuhu Bah, Amara Bangali Sesay, Stephen Sevalie, Joseph Sam Kanu

**Affiliations:** 1 Faculty of Basic Medical Sciences College of Medicine and Allied Health Sciences University of Sierra Leone, Freetown, Sierra Leone; 2 Institute for Global Health and Development, Queen Margaret University Edinburg, Musselburgh, Scotland, UK; 3 Faculty of Pharmaceutical Sciences, College of Medicine and Allied Health Sciences, University of Sierra Leone, Freetown, Sierra Leone; 4 Australian Research Centre in Complementary and Integrative Medicine, School of Public Health, Faculty of Health, University of Technology Sydney, Sydney, New South Wales Australia; 5 Directorate of Drugs and Medical Supplies Ministry of Health and Sanitation, Freetown, Sierra Leone; 6 34 Military Hospital Wilberforce, Freetown, Sierra Leone; 7 Directorate of Disease Prevention and Control, Ministry of Health and Sanitation, Freetown, Sierra Leone

**Keywords:** Anxiety, Depression, Post-traumatic stress disorder, Ebola, Ebola survivor, Sierra Leone

## Abstract

**Background:**

There is limited data available on the long-term mental health impact of Ebola virus disease (EVD) on survivors despite the disease experience of survivors meeting the criteria of a traumatic event as defined in the Diagnostic and Statistical Manual of Mental Disorders version IV (DSM IV). This study aimed to assess the prevalence and predictive factors of anxiety, depression and posttraumatic stress disorder among EVD survivors, approximately 2 years after discharge from the Ebola treatment centre (ETC).

**Methods:**

We conducted a cross-sectional study between May and August 2017 among 197 adults Ebola survivors in Bombali district, Northern Sierra Leone. We collected information about demographics, mental health status and possible predictive factors. The HAD scale was used to measure anxiety and depression. PTSD was measured using the PTSD-checklist (PCL). Chi-square test or Fisher exact two-tailed tests were used to test for associations and the multiple logistic regressions model to determine factors that were independently associated with the outcome variables.

**Results:**

The mean anxiety, depression and PTSD scores were (5.0 ± 3.9), (7.1 ± 3.8) and (39.5 ± 6.4) respectively. Based on cut-off scores, the prevalence of anxiety (HADs score ≥ 8), depression (HADs score ≥ 8) and PTSD (PCL ≥ 45) among Ebola survivors were (*n* = 49, 24.9%), (*n* = 93, 47.2%) and (*n* = 43, 21.8%) respectively. Older Ebola survivors (≥30 years) were more likely to show symptoms of depression (AOR = 8.5, 95% CI: 2.68–27.01, *p* = 0.001) and anxiety (AOR = 3.04; 95%CI: 1.2–7.7, *p* = 0.019) compared to younger ones (< 30 years). In addition, Ebola survivors who experienced a decreased level of exercise post-ETC discharge were more likely to show symptoms of depression (AOR = 2.63; 95%CI: 1.25–5.54, *p* = 0.011) and anxiety (AOR = 3.60; 95%CI: 1.33–9.72, *p* = 0.012) compared to those whose exercise remained the same post-ETC discharge.

**Conclusion:**

Our findings show that anxiety, depression and PTSD are common among the Ebola survivors in Bombali district, Northern Sierra Leone, and that underscores the need to diagnose and manage mental health morbidities among Ebola survivors long after their recovery from Ebola virus disease. Cognitive Behaviour Therapy (CBT) and Interpersonal Therapy (IPT) need to be explored as part of overall mental healthcare package interventions.

## Background

The West African Ebola virus disease (EVD) outbreak was the largest and worst compared to all previous outbreaks combined since it was discovered in 1976 [1]. It recorded an average case fatality rate of 51%, ranging from 41% in Sierra Leone to 61% in Guinea [2]. This resulted in 28,616 cases, 11,310 deaths and over 10,000 survivors across the three worst affected countries (Sierra Leone, Liberia and Guinea) [3]. Sierra Leone recorded 8704 infected cases, 3589 deaths, including 221 health workers [4]. It is estimated that a little over 4000 survived the EVD, many of which are experiencing post-Ebola physical and mental health sequelae [5–8].

The psychosocial consequences of the 11-year civil war (1991–2002) in Sierra Leone identified the need for mental health services. It also created an increased interest in mental health among researchers, policymakers, service users and carers, religious bodies, non-governmental organisations, civil society and healthcare providers. This lead to the establishment of mental health coalition in 2011 [9] and the enactment of the Mental Health Policy in 2012, which serves as a framework for the development of mental health services [10]. Despite this progress, access to quality mental health services is still a challenge as there are health system and community barriers that limit the delivery of effective mental health service [11]. Currently, Sierra Leone has two psychiatrists, two Clinical Psychologists and 19 mental health nurses that provide service to 7 million population [12]. Although the recent Mental Health Strategic Plan 2014–2018 provides a roadmap for the development of mental health curriculum, mental health course at tertiary health training institutions are still relatively non-existence [13]. The recent Ebola epidemic and mudslide further highlighted the dire need for mental health services considering the trauma affected communities (Ebola and mudslide survivors) were exposed to during and after such a devastating health and disaster emergencies respectively [5, 14]. A study in Western Urban and Western Rural districts of Sierra Leone indicated that EVD risk behaviours were significantly associated with symptoms of depression and post-traumatic stress disorder (PTSD) in the general population [15]. A nationwide survey also found that anxiety, depression and PTSD symptoms were prevalent in the general population a year after the end of the EVD response [16]. With the care of Ebola survivors integrated within the existing free health care program for pregnant women, lactating mothers and under-five children [17], it is expected Ebola survivors will have increased access to health services including mental health. To provide better mental health services to Ebola survivors, an understanding of the mental health status and their needs is crucial in informing policy and practice, which will improve resources to address mental health service gaps in Sierra Leone.

A review of the literature suggests that several sociodemographic and lifestyle characteristics have been shown to be associated with anxiety, depression and PTSD. For example, high rates of anxiety and depression were reported among females than males [18–20]. Also, low physical activity has been reported to be associated with an increased rate of anxiety and depression [21, 22]. In addition, studies have reported an association between increased alcohol intake and the development of anxiety and depressive symptoms among the general and sub-health populations [23–25].

Despite the disease experience of EVD survivors meeting the criteria of a traumatic event as defined in the DSM IV [26], there is a paucity of data with regards the long-term mental health impact among survivors. Several studies reported varying degree of psychiatric morbidity (depression, anxiety and post-traumatic stress disorder) among survivors in Guinea [27, 28], Liberia [29] and Sierra Leone [30–32]. Most of these studies were conducted at the peak of the outbreak using brief symptom-based tools and small sample sizes. Also, the Sierra Leonean studies failed to determine the socio = demographic and health-related correlates of the symptoms of anxiety, depressions and posttraumatic disorder. Thus, the aim of this study was to determine the prevalence and predictive factors of anxiety, depression and posttraumatic stress disorder among EVD survivors 2 years after discharge from an Ebola treatment centre (ETC).

## Method

### Study design and settings

We conducted a cross-sectional study among Ebola survivors residing in the Bombali district, Northern Sierra Leone, who visit the Bombali EVD Survivor Care Clinic. The Bombali EVD, Survivor Care Clinic, was established in October 2015 as a result of a partnership among the following institutions-Direct Relief, Medical Research Centre (MRC), and University of Makeni (UNIMAK), the Sierra Leone Ministry of Health and Sanitation (MoHS) and the Bombali EVD Survivor Association. The clinic provides outpatient services to survivors residing in the Bombali district and its environs. The clinic also provides referral services if needed to specialised care, at tertiary public health facilities within and outside the district. The services provided in the clinic include but are not limited to eye health, mental health awareness raising and sensitisation about Ebola survivorship.

### Sociodemographic characteristics and other Ebola-related variables

The sociodemographic characteristics include age (≤ 30 years, 30–49 years and > 50 years) sex (male / female), level of education (No formal education, primary, secondary and tertiary) marital status (single, married, divorced and widowed) and monthly income (< Le500, 000, Le 500,000– Le1, 000,000 and > Le1000, 000). Ebola-related variables explored include their past medical history of psychiatric illness prior to being diagnosed of Ebola (yes/no), the effect of the death of friend or relative (Not affected, slightly affected, badly affected and not applicable), number of current post-Ebola sequelae (none, 1–2 and > 2) and number of emotional support (none, 1–2 and > 2), knowledge of a relative or friend who died of Ebola (yes/no), alcohol status before (yes/no) and after Ebola (yes/no) and level of exercise after Ebola (increased, decreased, remain the same, not applicable) and alcohol intake after Ebola (increased, decreased, remain the same, not applicable).

### Main outcomes measures

Anxiety and depression among Ebola survivors were assessed using the Hospital Anxiety and Depression (HADS) Scale. The (HADS) Scale is a 14-item scale used to measure symptoms of anxiety and depression in the hospital, primary healthcare facility and among the general population [33]. The choice to use HADS scale was informed by the fact that it has been used to measure anxiety and depression among survivors of a similar post-infectious disease outbreak such as Severe Acute Respiratory Syndrome (SARS) [34]. The HADS scale has previously been used in Sierra Leone among ex-Ebola Treatment Centre (ETC) staff [35]. The HADS scale consists of seven items for the anxiety subscale and seven for the depression subscale. The anxiety subscale focused on symptoms of generalised anxiety disorder, and the depression subscale focused on anhedonia, which is considered the main symptom of depression [36]. Ebola survivors were asked to tick a particular response that they think is closest to how they have been feeling in the past week. Each of the 14 items was scored on a response scale with four alternatives that ranged from 0 to 3. The total score for each participant ranges from 0 to 21. Higher scores implied higher anxiety and depression levels. For both anxiety and depression, we used the recommended cut off score of 8 and above according to a clinically tested classification of psychiatric morbidity [33, 37, 38]. A score of ≥8 indicates a possible case of anxiety and depression, [33, 37, 38]. The HADS scale presents high internal consistency; Cronbach’s alpha coefficient was 0.884 (0.829 for anxiety and 0.840 for depression) and stability (test-retest intra-class correlation coefficient 0.944) with high concurrent validity [33]. Permission to use the HADS scale was obtained from GL Assessment Limited (Supplementary file 1).

The PTSD- checklist (PCL), a 17-item self-report measure of the 17 Diagnostic and Statistical Manual of Mental Disorders, Fourth Edition (DSM-IV) symptoms of PTSD was used to assess their level of PTSD among Ebola survivors. The PCL has been used to screen individuals for PTSD, diagnose PTSD, and monitor symptom change during and after treatment [39]. Intrusion, avoidance, and hyperarousal were considered as the primary domains of measurement with a higher internal consistency coefficient (Cronbach’s alpha of 0.939). Respondents indicate how much they have been bothered by a symptom over the past month using a 5-point (1–5) Likert scale. Responses ranged from 1 (Not at All) to 5 (extremely). We calculated the total symptom severity score (range = 17–85) for each participant [40]. A total score (45–85) was considered as a possible case of PTSD. A higher cut-off point score was used to identify definite cases and to minimise false positives [40]. The index trauma for PTSD in our study was the time of EVD diagnosis, and the time since the index trauma occurred to the time of data collection between 18 and 36 months. The PCL has been used in Sierra Leone among ex-Ebola Treatment Centre (ETC) staff [35] and other West African populations [41]. We did not obtain permission to use this tool given that this tool is available freely online and not copyrighted [42].

### Patient population and sampling method

A convenience sampling method was employed to recruit Ebola survivors into our study. An Ebola survivor was defined in our study as an adult (18 years and older) with a confirmed positive result on RT-PCR testing for Ebola virus on any body fluid who was treated successfully and discharged and with an EVD discharge certificate [43]. Ebola survivors attending the clinic during the period of data collection were recruited into the study. EVD survivor status was confirmed by comparing the clinic’s register with the Sierra Leone Association of Ebola Survivors registry of patients residing in the Bombali District and its environs, regardless of where they were originally treated. We excluded survivors who had severe physical or mental sequelae such as earing problems that limit their participation in the study.

### Data collection procedure

The data collection period was between May and August 2017, and it was done either by face-to-face interview in Krio (widely spoken language in Sierra Leone) for those who were illiterate or through self-administration of the anxiety, depression and PTSD instruments (for those who were literate). We initially piloted anxiety, depression and PTSD instruments among 10 EVD survivors whose data were excluded in the final data analysis. We explored the possibility of using self-administered and interviewer-administered formats of data collection during the piloting of the study instruments. No significant difference was observed between the two data collection procedures based on the feedback we received. To ensure that we collect accurate information using both data collection formats, the self-administered format was only allowed for survivors with tertiary education who indicated their willingness to answer the questions on their own.

We also sent the study instruments to public health, infectious disease and mental health experts for review. This was followed by group discussions among researchers, linguistic expert and data collectors to reach a consensus in a bid to minimise error during the data collection process. We trained two pharmacy professionals, a community health officer and a nurse from the Bombali District Health Management Team (DHMT) as data collectors on the overall research protocol, informed consent, confidentiality, data collection techniques and quality assurance. We held a stakeholder meeting with the management of the clinic and the DHMT, where we explained the full protocol of the research. The clinic coordinator approached adults Ebola survivors attending the clinic during the period of data collection. EVD survivors were invited to take part in the study after explaining to them about the research. EVD survivors were taken through the participant information sheet, the informed consent form and those that consented and signed or thumb-printed the informed consent form were interviewed. Participants were informed of their right to opt-out of the study at any point if they wish to discontinue without any explanation, and that they would not receive direct benefit from participating, other than the psychosocial support they will receive for those that score above the threshold.

### Ethical clearance

The Sierra Leone Ethics and Scientific Review Committee, Ministry of Health and Sanitation gave ethical approval before the start of the study. Written informed consent was obtained from participant either by signing or thumb printing (illiterate participants) the consent form.

### Data analysis

Data analysis was done using SPSS Package version 24 (SPSS, Inc.; Chicago). Descriptive statistics such as means and standard deviation as well as frequency and percentages were used to report continuous and categorical variables respectively. Chi-square or Fisher exact was used to test for associations between the independent variables (age, sex, marital status, number of emotional supports etc) and outcome variables (anxiety, depression and post-traumatic stress disorder). In the univariate, bivariate and multivariate analysis, anxiety, depression and PTSD scores were converted into binary variable based on their respective cut-off scores. A score of < 8 was considered as no possible case of anxiety and depression. A score ≥ 8 was considered as a possible case of anxiety and depression. With regards to PTSD, a score < 45 was considered as no possible case of PTSD. A score ≥ 45 was considered as a possible case of PTSD. Independent variables, i.e. demographic and health-related variables with *p*-value ≤0.25 in the bivariate analysis, i.e. Chi-square or Fisher exact test, were entered into the univariate model in order to determine the odd crude ratio. Independent variables with *p* ≤ 0.05 were then entered into the multivariable logistic regression model to determine factors that were independently associated with outcome variables (anxiety, depression and PTSD). Statistical significance was defined as a two-tailed, *p*-value less than 0.05 for all inferential analysis.

## Results

### Sociodemographic characteristics of Ebola survivors

Of 245 Ebola survivors approached, 197 agreed to participate in the study giving a response rate of 80.4%. Table 1 summarises the sociodemographic characteristics of Ebola survivors. Majority (*n* = 167, 84.8%) were below the age of 50 years, and half (*n* = 99, 50.3%) of them were females. Close to half (*n* = 84, 42.6%) were single. More than two-third (*n* = 140, 71.1%) were badly affected as a result of the death of a friend or a relative due to Ebola, whereas the exercise level among more than half (*n* = 110, 55.8%) of survivors decreased after recovery. Our study adheres to the STROBE guidelines for cross-sectional studies. Please see supplementary file 2 for details.

**Table 1:** demographics of Ebola survivors (*n* = 197)

Characteristics	Variables	N (%)
Age Group	< 30 years	87(44.2)
	30-49 years	80(40.6)
	≥50 years	30(15.2)
Sex	Male	98(49.7)
	Female	99(50.3)
Marital status	Single	84(42.6)
	Married	59(29.9)
	Divorced	13(6.6)
	Widow/widower	41(20.8)
Monthly income level	< Le500, 000	141(71.6)
	Le 500,000– Le1, 000,000	52(26.4)
	>Le1000, 000	4(2.0)
Educational status	Non-formal education	73(37.1)
	Primary education	32(16.2)
	Secondary education	68(34.5)
	Tertiary education	24(12.2)
Number of emotional support	None	7(3.6)
	1–2	70(35.5)
	> 2	120(60.9)
Effect of death of friend or relative	Not affected	9(4.6)
	Slightly affected	29(14.7)
	Badly affected	140(71.1)
	Not applicable	19(9.6)
Number of current post-Ebola sequelae	None	22(11.2)
	1–2	45(22.8)
	> 2	130(66.0)
Past psychiatric history prior to being diagnosed with Ebola	Yes	8(4.1)
	No	189(95.9)
Alcohol intake before Ebola	Yes	60(30.5)
	No	137(69.5)
Alcohol intake after Ebola	Increased	12(6.1)
	Decreased	31(15.7)
	Remain the same	23(11.7)
	Not applicable	131(66.5)
Level of exercise after Ebola	Increased	33(16.8)
	Decreased	110(55.8)
	Remain the same	50(25.4)
	Not applicable	4(2.0)
Knowledge of relative or friend death due to Ebola	Yes	179(90.9)
	No	18(9.1)

### Prevalence of anxiety, depression and PTSD

Table 2 presents the summary statistics of the anxiety, depression and PTSD. The mean anxiety, depression and PTSD scores were (5.0 ± 3.9), (7.1 ± 3.8) and (39.5 ± 6.4) respectively. Table 2 also indicates that close to a quarter (*n* = 49, 24.9%), close to half (*n* = 93, 47.2%) and close to a quarter (*n* = 43, 21.8%) of survivors interviewed showed a possible case of anxiety, depression and PTSD respectively.

**Table 2:** Prevalence of anxiety, depression and PTSD

**Anxiety scale**	Mean (SD)	5.0 (3.9)
	Possible case of anxiety (HADS cut-off score ≥ 8) n (%)	49(24.9)
**Depression scale**	Mean (SD)	7.1(3.8)
	Possible case of depression (HADs cut-off score ≥ 8) n (%)	93(47.2)
**PTSD scale**	Mean (SD)	39.5(6.4)
	Possible case of PTSD (cut-off score ≥ 45) n (%)	43(21.8)

### Association between anxiety, depression, PTSD and demographic variables

Table 3 highlights the association between anxiety, depression, PTSD and the demographic variables. Age, death of friend or relative, alcohol intake before Ebola, level of exercise after Ebola and knowing a relative or a friend that died due to Ebola was associated with the symptoms of anxiety among survivors. Number of emotional supports received age, the number of post-Ebola symptoms experienced, alcohol intake before and after Ebola and level of exercise after Ebola were associated with symptoms of depression among survivors. However, no sociodemographic and health-related characteristics were associated with symptoms of PTSD among survivors.

**Table 3:** Association between anxiety, depression, PTSD and demographic and health- related variables

		Anxiety			Depression			PTSD		
Characteristics	Variables	Possible case of anxiety (HADS cut-off score ≥ 8) Symptom of anxiety n(%)	No possible case of anxiety (HADS cut-off score < 8) n (%)	*P*-value	Possible case of depression (HADs cut-off score ≥ 8)	No possible case of depression (HADs cut-off score < 8)	*P*-value	Possible case of PTSD (cut-off score ≥ 45)	No possible case of PTSD (cut-off score < 45)	*P*-value
Age Group	< 30 years	34(69.4)	133(89.9)	0.001	67(72.0)	100(96.2)	< 0.001	36(83.7)	131(85.1)	0.828
	≥30 years	15(30.6)	15(10.1)		26(28.0)	4(3.8)		7(16.3)	23(14.9)	
Sex	Male	25(49.3)	73(51.0)	0.837	47(50.5)	51(49.0)	0.834	21(48.8)	77(50.0)	0.893
	Female	24(49.0)	75(50.7)		46(49.5)	53(51.0)		22(51.2)	77(50.0)	
Marital status	Single /divorced/widow/widower	36(73.5)	102(68.9)	0.547	63(67.7)	75(72.1)	0.503	32(74.4)	106(68.8)	0.479
	Married	13(26.5)	46(31.1)		30(32.3)	29(27.9)		11(25.6)	48(31.2)	
Monthly income level	< 1000,000 Leones	48(98.0)	145(98.0)	0.995	90(96.8)	103(99.0)	0.261	42(97.7)	151(98.1)	0.877
	≥1million	1(2.0)	3(2.0)		3(3.2)	1(1.0)		1(2.3)	3(1.9)	
Educational status	Non-formal education	17(34.7)	56(37.8)	0.558	39(41.9)	34(32.7)	0.578	18(41.9)	55(35.7)	0.576
	Primary education	6(12.2)	26(17.6)		13(14.0)	19(18.3)		4(9.3)	28(18.2)	
	Secondary education	21(42.9)	47(31.8)		30(32.3)	38(36.5)		16(37.2)	52(33.8)	
	Tertiary education	5(10.2)	19(12.8)		11(11.8)	13(12.5)		5(11.6)	19(12.3)	
Number of emotional support	None	3(6.1)	4(2.7)	0.243	4(4.3)	3(2.9)	0.038	2(4.7)	5(3.2)	0.459
	1–2	20(40.8)	50(33.8)		41(44.1)	29(27.9)		12(27.9)	58(37.7)	
	> 2	26(53.1)	94(63.5)		48(51.6)	72(69.2)		29(67.4)	91(59.1)	
Affect by the death of friend or relative	Not affected	5(10.2)	4(2.7)	0.001	6(6.5)	3(2.9)	0.528	3(7.0)	6(3.9)	0.533
	Slightly affected	3(6.1)	26(17.6)		15(16.1)	14(13.5)		4(9.3)	25(16.2)	
	Badly affected	31(63.3)	109(73.6)		62(66.7)	78(75.0)		31(72.1)	109(70.8)	
	Not applicable	10(20.4)	9(6.1)		10(10.8)	9(8.7)		5(11.6)	14(9.1)	
Number of current post-Ebola sequelae experienced	None	6(12.2)	16(10.8)	0.274	11(11.8)	11(10.6)	0.021	2(4.7)	20(13.0)	0.337
	1–2	15(30.6)	30(20.3)		29(31.2)	16(15.4)		10(23.3)	35(22.7)	
	> 2	28(57.1)	102(68.9)		53(57.0)	77(74.0)		31(72.1)	99(64.3)	
Past psychiatric history prior to being diagnosed with Ebola	Yes	4(8.2)	5(3.4)	0.164	4(4.3)	5(4.8)	0.865	2(4.7)	7(4.5)	0.977
	No	45(91.8)	143(96.6)		89(95.7)	99(95.2)		41(95.3)	147(95.5)	
Alcohol intake before Ebola	Yes	21(42.9)	39(26.4)	0.030	38(40.9)	22(21.2)	0.003	18(41.9)	42(27.3)	0.066
	No	28(57.1)	109(73.6)		55(59.1)	82(78.8)		25(58.1)	112(72.7)	
Alcohol intake after Ebola	Increased	3(6.1)	9(6.1)	0.333	6(6.5)	6(5.8)	0.020	4(9.3)	8(5.2)	0.310
	Decreased	11(22.4)	20(13.5)		20(21.5)	11(10.6)		8(18.6)	23(14.9)	
	Remain the same	7(14.3)	16(10.8)		15(16.1)	8(7.7)		7(16.3)	16(10.4)	
	Not applicable	28(57.1)	103(69.6)		52(55.9)	79(76.0)		24(55.8)	107(69.5)	
Level of exercise after Ebola	Increased	5(10.2)	28(18.9)	0.010	11(11.8)	22(21.2)	0.002	8(18.6)	25(16.2)	0.575
	Decreased	37(75.5)	73(49.3)		64(68.8)	46(44.2)		26(60.5)	84(54.5)	
	Remain the same	6(13.0)	44(29.7)		18(19.4)	36(34.6)		9(20.9)	45(29.2)	
	Not applicable	1(2.0)	3(2.0)		0(0.0)	0(0.0)		0(0.0)	0(0.0)	
Knowledge of relative or friend death due to Ebola	Yes	41(83.7)	138(93.2)	0.044	84(90.3)	95(91.3)	0.803	41(95.3)	138(89.6)	0.248
	No	8(16.3)	10(6.8)		9(9.7)	9(8.7)		2(4.7)	15(10.4)	

### Predictors of anxiety, depression and PTSD among Ebola survivors

Tables 4 and 5 indicate that older Ebola survivors (≥30 years) were more likely to show symptoms of depression (AOR = 8.5, 95%CI: 2.68–27.01, *p* = 0.001) and anxiety (AOR = 3.04; 95%CI: 1.2–7.7, *p* = 0.019) compared to younger ones (< 30 years). In addition, Ebola survivors who experience a decreased level of exercise post-ETC discharge were more likely to show symptoms of depression (AOR = 2.63; 95%CI: 1.25–5.54, *p* = 0.011) and anxiety (AOR = 3.60; 95%CI: 1.33–9.72, *p* = 0.012) compared to those whose exercise remained the same post-ETC discharge. On the other hand, Table 6 shows that none of the sociodemographic variables considered in our study was a predictor of PTSD among survivors.

**Table 4:** Predictors of anxiety among Ebola survivors

Characteristics	Variables	OR (95%CI)	*P*-value	Adjusted OR (95%CI)	*P*-value
Age Group	< 30 years	1	**0.001**	1	**0.019**
	≥30 years	3.91(1.74–8.78)		**3.04**(1.20–7.70)	
Number of emotional support	None	1		–	
	1–2	0.53(0.11–2.60)	0.437	–	
	> 2	0.37(0.08–1.75)	0.210	–	
Effect of death of friend or relative	Not affected	1		1	
	Slightly affected	0.09(0.02–0.55)	**0.009**	0.04(0.01–0.32)	**0.002**
	Badly affected	0.23(0.06–0.90)	**0.035**	**0.12**(0.03–0.58)	**0.008**
	Not applicable	0.89(0.18–4.38)	0.885	**0.51**(0.09–3.03)	0.462
Past psychiatric history	No	1	0.178	–	
	Yes	2.54(0.66–9.87		–	
Alcohol intake before Ebola	No	1	**0.031**	1	
	Yes	2.10(1.07–4.11)		1.93(0.85–4.35)	0.115
Level of exercise after Ebola	Remains the same	1		1	
	Increased	1.20(0.35–4.14)	0.774	1.30(0.34–4.50)	0.700
	Decreased	3.40(1.40–8.26)	**0.007**	**3.60**(1.33–9.72)	**0.012**
Knowledge of relative or friend death due to Ebola	No	1	0.051	–	–
	Yes	0.37(0.14–1.00)		–	–

**Table 5:** Predictors of depression among Ebola survivors

Characteristics	Variables	OR (95%CI)	*P*-value	Adjusted OR(95%CI)	P-value
Age Group	< 30 years	1	**< 0.001**	1	**0.001**
	≥30 years	9.70(3.24–29.06)		8.51(2.68–27.01)	
Number of emotional support	None	1		–	
	1–2	1.06(0.22–5.10)	0.942	–	
	> 2	0.50(0.11–2.33	0.378	–	
Number of sequelae experienced	None	1		–	
	1–2	1.81(0.64–5.10)	0.260	–	
	> 2	0.69(0.28–1.70)	0.419	–	
Alcohol intake before Ebola	No	1	**0.003**	1	
	Yes	2.58(1.38–4.82)		0.10(0.24–4.21)	0.998
Alcohol intake after Ebola	Increased	1.52(0.47–4.97)	0.489	2.68(0.47–15.18)	0.265
	Decreased	2.76(1.22–2.64)	**0.015**	1.63(0.36–7.43)	0.531
	same	2.85(1.13–7.20)	**0.027**	1.67(0.32–8.83)	0.546
	Not drinking	1		1	1
Level of exercise after Ebola	Remains the same	1		1	
	Increased	1.00(0.40–2.51)	1.00	0.84(0.29–2.43)	0.747
	Decreased	2.78(1.41–5.50)	**0.003**	2.63(1.25–5.54)	**0.011**

**Table 6:** Predictors of Post-traumatic stress disorder (PTSD) among Ebola survivors

Characteristics	Variables	OR (95%CI)	*P*-value	Adjusted OR	*P*-value
Alcohol intake before Ebola	No	1			
	Yes	1.92(0.95–3.87)	0.069	**–**	
Knowledge of relative or friend death due to Ebola	No	1			
	Yes	2.38(0.53–10.77)	0.261	**–**	

## Discussion

Several studies have reported the prevalence of symptoms of psychological distress among Ebola survivors immediately after being infected or shortly after discharge from the Ebola treatment centre [5, 6]. However, there is paucity of data on the long-term mental health effects among those who survived Ebola virus disease. This is the first ever-quantitative study that assessed the long-term prevalence and predictors of the symptoms of anxiety, depression and post-traumatic stress disorder among Ebola survivors in Sierra Leone. Our study is aimed to provide an insight into the long-term psychological morbidity and its possible risk factors among Ebola survivors in Sierra Leone in order to inform ongoing policy and practice related to providing care to Ebola survivors.

Our study indicates that close to a quarter (24.9%) of survivors exhibited a possible case of anxiety which is slightly higher than those reported by Nanyonga et al. [31] and Mohamed et al. [44] in Kenema, Eastern Sierra Leone. Although studies from East Africa [45–47] and in Guinea [28], Nigeria [48] Liberia [49] and within Sierra Leone [30, 50–52] using quantitative and qualitative research designs have reported that psychological harm is common among Ebola survivors, it has failed to assess anxiety among Ebola survivors specifically, and this makes comparison among studies to be difficult. For instance, a study by Ji et al. in Sierra Leone, anxiety was part of positive symptom distress index (PSDI) that was used to evaluate psychological distress among Ebola survivors, medical personnel and logistics workers [51]. In reporting, only the PSDI score was reported, and it was higher among Ebola survivors compared to non-survivors [51].

With regards to depression, our study indicates that close to half (47.2%) of Ebola survivors interviewed show possible case of depression. Our result is slightly above a similar study conducted in Liberia among 82 survivors, 6 months following their discharge from ETC [53] It is also higher than the prevalence reported among survivors in eastern Sierra Leone (35%) that were assessed 4 months post-ETC discharge [31].. In addition, a cohort study in Guinea among survivors most of which were assessed 8 months post-ETC discharge, reported a lower prevalence of depressive symptoms (15%) although three, thirteen and eleven of the 33 survivors who were assessed by a psychiatrist show mild, moderate and severe depression respectively [54]. Self-perceived depression was reported in 1% of survivors in another Guinean study [27]. The variation observed between our finding with that of other findings from other studies may be due to differences in the post ETC discharge time when Ebola survivors were assessed. It may also be due to the use of different evaluation tools to assess depression as well as the varied sociocultural dynamics and coping mechanisms employed by survivors in each of the study areas.

Post-traumatic stress disorder is a mental disorder that is common among individuals that have experienced severe trauma such a war, sexual violence or deadly infectious disease outbreak such as EVD. Our study reports that close to a quarter (21%) of EVD survivors interviewed, showed a possible case of PTSD. A similar prevalence of post-traumatic stress reactions three-four weeks post-ETC discharge which is predictive of PTSD was reported in a study by Hugo and colleagues in Kailahun-Eastern Sierra Leone [30]. The reason for such similarity in our findings may be due to coincidence. Future nationwide studies should explore whether our findings hold true in other parts of Sierra Leone and other Ebola-affected countries. Compared to other infectious disease outbreaks, in our study, our PTSD prevalence is slightly lower than that reported on SARS survivors in Hong Kong [34]..

Our study revealed that symptoms of anxiety and depression were more common among survivors above the age of 30 years and whose rate of exercise has decreased after surviving Ebola. Low physical activity was found to be associated with anxiety and depression among chronic disease patients in Myanmar and Vietnam [22]. Physical activity should be part of mental health promotion interventions given that increase physical activity and low sedentary behaviour are known to improve depressive [21, 55] and anxiety [56] symptoms in survivors of other chronic disease conditions. In addition to physical exercise, evidence-based mental health interventions such as Behavioural Activation (BA) need to be explored as part of mental health care interventions given that BA- as part of the Youth Readiness Intervention (YRI) - has been proven to be effective in the treatment of symptoms of common mental disorders such as depression among war-affected youths in Sierra Leone. Although further research into our reported associations is needed, our findings suggest that being old and decreased physical activity, which might be due to the widely reported musculoskeletal complications among EVD survivors [7] may be specific risk factors for anxiety and depression among Ebola survivors and may warrant early and focused support services. Even though studies have reported a positive correlation between increased alcohol intake and high rates of anxiety and depression [23–25], results from our regression analyses did not find alcohol intake as a statistically significant predictor of anxiety, depression and PTSD among Ebola survivors. Differences in sample population, socio-economic and cultural context as well as in the use of study instruments may help explain why there were no significant associations between alcohol intake and anxiety, depression as well as PTSD. Further large-scale studies are required to ascertain whether a positive association between alcohol intake and anxiety, depression as well as PTSD.

We observed in our study that none of the sociodemographic variables considered is a possible predictor of symptoms of post-traumatic stress disorder experienced by survivors although a study among Severe Acute Respiratory Syndrome (SARS) survivors reported that being female and the presence of chronic health condition diagnosed before being infected with SARS were predictors of PTSD among SARS survivors in Hong Kong [57]. It is worthy to note that even though the general literature suggests that the rates of depression and anxiety can be higher among females than males [18–20, 58, 59], analysis of our data suggest that there is no statistically significant association between the rates of depression and anxiety and gender in our study (see Table 3). Reasons for this may be due to differences in the traumatic event that triggered their psychological distress, in this case, being infected with the Ebola virus. It may also be due to differences in resilience/ coping mechanism developed in responding to the traumatic event. Another reason maybe linked to the sample size used and the fact that a non-probability sampling method was used in our study.

### Policy and clinical implications and future direction

In light of the relatively high prevalence of symptoms of anxiety, depression and PTSD, the care for Ebola survivors should not only focus on the treatment of physical conditions. Healthcare workers providing care to survivors should be aware of the possible long-term psychological complications, especially symptoms of anxiety, depression and PTSD. As it has been reported in previous studies among the general population in Sierra Leone [15, 16], our study further highlights the significance of detecting and managing psychiatric morbidity among survivors following a devastating and traumatic event or an infectious disease outbreak such as Ebola.

For future outbreaks or other traumatic events, the design and execution of response during and after the outbreak should factor in the high possibility of the increasing incidence of mental health morbidities as it has been observed in other infectious disease outbreaks [60]. The training of mental health staff and the integration of mental health into the existing primary healthcare system will contribute to improving access to psychosocial care in a country where access to mental health care is limited. Psychosocial support with self-copying strategies is needed to enhance survivors’ resilience in dealing with anxiety, depression and PTSD. The application of disaster response management knowledge and skills can be useful while awaiting further research evidence on the effectiveness of psychosocial interventions given the fact that Ebola shares a similar clinical pattern of psychiatric morbidity with other disasters. The Cognitive-behavioural therapy (CBT) is a mental health intervention that can be used to manage psychiatric morbidity among survivors in Sierra Leone given that previous Sierra Leonean studies have reported the effectiveness of CBT in improving the mental health, social behaviour, and school functioning among war-affected youth [61] and ex-Ebola Treatment Centre staff [35]. Also, the use of group interpersonal therapy (IPT) needs to be explored as it was effective in treating depressive symptoms in Uganda [62] and South Africa [63].

Future longitudinal cohort studies are required to understand the trajectory of mental health morbidities among Ebola survivors. Multicentre or cross-country studies are needed to unearth any cultural differences that may exist and come up with findings that are applicable to a wider population. The psychological impact of Ebola on at risk populations such as pregnant women, children, immediate relatives of Ebola survivors and survivors of recent natural disaster in Sierra Leone is an area worth exploring.

### Strengths and limitations

To the best of our knowledge, this is the first study that has explored the long-term psychological complications among Ebola survivors in Sierra Leone. However, the interpretation of our findings should be done with caution, considering the following limitations. Our findings are only representative of Bombali district and not the whole of Sierra Leone. Although we used widely used pre-validated instruments to measure anxiety, depression and PTSD, none of them was validated in Sierra Leone before the conduct of our study. As such, we were unable to validate the clinical cut-off point for anxiety, depression and PTSD among the Ebola survivors that participated in our study. Instead, we used clinical cut-off points based on the available literature [33, 36, 37, 40]. In addition, comparison of our findings with others was difficult since, to the best of our knowledge, these studies used different tools to measure symptoms of anxiety, depression and PTSD among the Ebola survivors. Our study was conducted without a control group, and that makes interpretation of our findings is little bit challenging. In our study, we rely on self-report to measure anxiety, depression and PTSD as well as retrospective reporting of some of the sociodemographic and lifestyle characteristics. As such, we cannot totally rule out the possibility of recall bias. We excluded Ebola survivors with hearing impairment, which may limit their ability to provide information accurately. Although the demographic characteristics of Ebola survivors excluded are similar to those included in our study, it is possible that those excluded may have provided different responses to questions asked in our study. The index trauma for PTSD in our study was the time of EVD diagnosis. However, we were unable to report the time since the index trauma occurred to the time of data collection for each Ebola survivors.

Owing to the cross-sectional design, the present study only described anxiety, depression and PTSD symptoms of Ebola survivors 2 years after the end of the Ebola outbreak in Sierra Leone. Longitudinal studies are required in order to monitor changes over time.

## Conclusion

Our study suggests that anxiety, depression and PTSD are common among Ebola survivors in Bombali district, Northern Sierra Leone. Also, our findings suggest that being above the age of 30 years and decreased rate of exercise after surviving Ebola are likely predictors of anxiety and depression but PTSD among Ebola survivors. Our study further underscores the importance of detecting and managing mental health morbidities among survivors after exposure to an infectious disease outbreak such as Ebola. Cognitive Behaviour Therapy (CBT) and Interpersonal Therapy (IPT) need to be explored as part of overall mental healthcare package interventions.

## Supplementary information

**Additional file 1: Supplementary file 1.** Countersigned User Agreement for the use of HADS. Countersigned user agreement for the use of HADS in this study between GL Assessment Limited and the Corresponding Author.

**Additional file 2: Supplementary file 2.** STROBE checklist for cross-sectional studies. Adherence to STROBE guidelines for cross-sectional studies.

## Data Availability

The datasets used for the current study are available from the corresponding author on reasonable request (abdulaijawobah@yahoo.com and jamepeb@yahoo.com).

## Abbreviations

AOR: Adjusted odd ratio
CBT: Cognitive-behavioural therapy
DHMT: District Health Management Team
DSM-IV: Manual of mental disorders, fourth edition
EVD: Ebola virus disease
ETC: Ebola treatment centre
HADS: Hospital Anxiety and Depression Scale
IPT: Interpersonal therapy
MoHS: Ministry of Health and Sanitation
PTSD: Post-traumatic stress disorder
SARS: Severe acute respiratory syndrome
WHO: World Health Organisation
