# Supplementary material for: Prevalence of anxiety, depression and post-traumatic stress disorder among Ebola survivors in northern Sierra Leone: a cross-sectional study
Source: BMC Public Health. 2020 Sep 11;20:1391. doi: 10.1186/s12889-020-09507-6 (PMC7485189; doi:10.1186/s12889-020-09507-6)
Supplement: Supplementary file 1 — Additional file 1: Supplementary file 1. Countersigned User Agreement for the use of HADS. Countersigned user agreement for the use of HADS in this study between GL Assessment Limited and the Corresponding Author. [file 12889_2020_9507_MOESM1_ESM.pdf]

GL Assessment Ltd

Hospital Anxiety and Depression Scale© (HADS)

PERMISSIONS REGISTRATION FORM AND USER AGREEMENT

Please note that the Terms and Conditions of this agreement are non-negotiable. Modifications to the agreement will not be accepted unless approved in writing by GL Assessment. Please read this agreement in full before completion, especially the notes section starting on Page 8.
Note: This agreement can be completed and returned electronically. **Please provide all details and return as a Word doc attachment**. Once all details are completed, please sign the agreement on page 7 and scan in the entire document and return to GL Assessment for countersignature.

Agreement Dated 12/08/2020

Section 1 – Licensee's contact details & full invoice address

LICENSEE : :Peter Bai James ***(note 1)***

Address : 29A Barcom Street , Merrylands West, Sydney)

Country : Australia]

Postcode/Zip [2160]

VAT Number (if applicable) : : ………………………………………………………………***(note 2)***

Contact Details:

Name : Abdulai Jawo Bah ..

Title :Lecturer Department of Pharmacology, College of Medicine and Allied Sciences (COMAHS), University of Sierra Leone (USL), Connaught Teaching Hospital, Perceval Street Freetown.

Phone :+23275131871 Fax :…………NA…..

Email :abdulaijawobah@yahoo.com.

Invoice Address if Different from above: (*credit account form is required for invoicing – **note 3***)

…………………………Same as above…………………………………………………………

Section 2 – Health, Social Care and Specialist Resources

Which of these bests describes your current organisation or institution's area/s of activity?

☐ NHS Trust
☐ Other Hospital
☒ University/College
☐ Health Authority
☐ Pharmaceutical
☐ Child Psychology and Mental Health
☐ Personal and Social Development
☐ Speech and Language
☐ Special Educational Needs (SEN)
☐ Neuropsychology

Section 3– Membership /Qualifications/ Training /Experience (if registering for the first time)

***Professional Membership**
Please list membership of professional bodies, including your registration or membership number

Pharmaceutical society of Sierra Leone, registration number 142

***Academic Qualifications**

Please give full details of qualification and subject. If none, please write 'none'. Note that the information you give here will determine which services you will be able to take advantage of, so please provide as much detail as possible.

Qualification	Subject	Institution	Date	Certificate encl.
Bachelor of Pharmacy	Pharmacy	University of Sierra Leone	December 2008	
Master of Medicine	Chinese Materia Medica (Pharmacology)	Tianjin University of traditional Chinese Medicine	June 2013	

*Relevant Further Training

e.g. Postgraduate Certificate in Education, psychology qualification. Please give full details of qualification and subject. If none, please write 'none'. **Please include a photocopy of your certificate or diploma with this form. Your registration cannot be processed without it.**

Previous experience:

Please give details of any health and psychology test instruments and/or health questionnaires you have used and whether you administered directly or under supervision. Again, please provide as much detail as possible.

I have used HIV related stigma for people living with HIV/AIDS instrument (HASI-P). The HASI-P is a validated 33-item scale that measures stigma among HIV/AIDS patients in the past 3 months [43]. This instrument was validated among HIV/AIDS patients in five African countries: Lesotho, Malawi, South Africa, Swaziland and Tanzania I adapted this tool in Sierra Leone to measure stigma among Ebola survivors, given that there are currently no validated tool to measure stigma among Ebola survivors. I administered it directly without any supervision.

I have also designed questionnaires on complementary and alternative medicine use among Ebola survivors, pregnant women, infertile women, lactating mothers and children under the age of five based

Section 4 – GL registration details (to be completed by GL if these details have not already previously been provided)

on the available literature. These questionnaires were piloted in order to appropriate to our local context. I administered these without supervision to the various targeted populations.

I have also designed and administered questionnaires relating to pharmacy students future career intentions. In addition I have adapted and self-administered a validated questionnaire that assesses knowledge about and attitude towards influenza vaccination.

Note: If you have already received your account details (Reader and Qualification Codes), please skip section 3 and go straight to section 4

GL ACCOUNT NUMBER – […208741…………………………………………………………………………………….]

GL READER CODE (Mandatory- *note 4*)…295098………………..

GL QUALIFICATION CODE (Mandatory – *note 4*)…PER…………………………………………….…………

*<u>For student licensees the following details are also required- (**note 5**):</u>*

University Course and supervisor's name : .... ……………....

………

Supervisor's GL Reader Code ....

Supervisor's GL Qualification Code..

Section 5 – Context of HADS use

PROJECT/STUDY NAME AND DETAILS (*note 6*): Study title: Prevalence and predictive factors of anxiety, depression and posttraumatic stress disorder among survivors of EVD and COVID19 pandemic in Sierra Leone

Study Summary: There is limited data available on the long-term mental health impact of survivors of emerging disease outbreaks such as Ebola virus disease (EVD) and the current COVID19 pandemic despite the disease experience of survivors meeting the criteria of a traumatic event as defined in the Diagnostic and Statistical Manual of Mental Disorders version IV (DSM IV). This study aimed to assess the prevalence and predictive factors of anxiety, depression and posttraumatic stress disorder among survivors of EVD and COVID19 pandemic in Sierra Leone. We intend to conduct a cross-sectional study among survivors of Ebola and COVID19 pandemic in Sierra Leone. We will use HADS and PCL-5 instruments to assess anxiety, depression and posttraumatic stress disorder among survivors of Ebola and COVID19 pandemic in Sierra Leone

STUDY GROUP

- Abdulai Jawo Bah, Peter Bai James, Nuhu Bah, Amara Bangali Sesay, Stephen Sevalie, and Joseph Sam Kanu

……

- **Number of expected study participants/subjects :** 150
- **Number of administrations of the questionnaire per participant/subject :** 1
- **TOTAL NUMBER OF ADMINISTRATIONS (*note 7*)** : 150
- **Planned study dates**: *start* August/ 2020 (month/year) *end* December 2020 (month/year)

Section 6 – Project/Study financing

Please indicate here if your use of HADS will be for commercial use, healthcare or academic research/non-commercial use.

For commercial use: ☐ Qty of administrations

Use by a registered company, an organisation, establishment or individual that enables them to, or is part of them benefitting monetarily by their research with the questionnaire, or the application of the questionnaire, CRO, pharmaceutical and any for-profit companies.

For Healthcare organisations/institutions: ☐ Qty of administrations

Classified as hospitals, General Practitioners, healthcare centres, sports and rehabilitation centres, research organisations, scientific societies and charities.

For academic research/non-commercial use: ☒ Qty of administrations 150

Classified as use by an individual or organisation that is using the questionnaire purely for research or study purposes without financial gain or use by an individual.

Fee for commercial use:
Price shown is per administration/use

0-1000 @ £1.35 each
1001-2500 @ £1.25 each
2501 + @ £1.15 each

Fee for Healthcare organisations/institutions:
Price shown is per administration/use

0-1000 @ £1.10 each
1001-2500 @ £1.00 each
2501 + @ £0.90 each

Fee for academic research/non-commercial use:
Price shown is per administration/use

0-1000 @ £0.90 each
1001-2500 @ £0.85 each
2501 + @ £0.80 each

*Please include cost of Manual at **£52.40 per copy (plus shipping)** in any quote:* *Yes* ☐ *No* ☒

Quantity required: ……………………………………

TOTAL ADMINISTRATIONS/COPYRIGHT FEE COSTS: £ 135]

TOTAL MANUAL/S COSTS: £[0]

TOTAL MANUALS SHIPPING COSTS (GL to add): £ [ ]

ADMINISTRATION FEE (if applicable): £[…Not applicable ***……………………………………]***

Please Note: An additional administration fee of £60.00 will be applicable to orders under £100.00 net in value.

TOTAL INVOICE VALUE: £135

Section 7 – HADS versions and translations (note 8)
(These will be sent to you as a PDF file)

DO YOU REQUIRE THE HADS IN UK ENGLISH? YES ☒ NO ☐

IF SO, WHICH VERSION; ORIGINAL TEMPLATE OR WORD VERSION? Word version…………..

PLEASE INDICATE HERE IF YOU WILL REQUIRE TRANSLATIONS (A separate translation agreement will be required between the LICENSEE and the Mapi Research Trust and is not part of this Agreement). If appropriate, please indicate in which language(s) and for which country(ies) the **HADS** is needed. See **Note 8** for Mapi contact details to obtain a list of available translations.

Do **NOT** include German for Germany, Austria or Switzerland. See **note 8** for further details of how to obtain this translation.

Please enter the required languages into the following table. If you require more fields than currently shown, place your cursor to the right of the last cell and press return.

Languages:

LICENCE AGREEMENT, made on the date of this document between GL Assessment Limited of 1st Floor Vantage London, Great West Road, Brentford TW8 9AG, United Kingdom (hereinafter called 'the Publisher') and the LICENSEE as defined on the first page of this agreement.

NOW IT IS MUTUALLY AGREED between the parties hereto as follows:

The Publishers hereby grants permission for the Licensee to reproduce in the printed format up to the TOTAL NUMBER OF ADMINISTRATIONS of the HOSPITAL ANXIETY AND DEPRESSION SCALE (HADS) ('the Material') subject to the following conditions to which the Licensee hereby agrees:

1. The Licensee agrees that it is only permitted to use the Materials for the purpose of the PROJECT/STUDY and on the terms set out in this User Agreement and Notes (collectively "the Agreement").

2. In consideration for the rights granted to the Licensee by this Agreement, the Licensee hereby agrees to pay to the Publisher the TOTAL COPYRIGHT FEE as defined above for the number of administrations detailed above. Further administrations over and above the specified amount may be negotiated as required on terms to be agreed. The Licensee shall pay such copyright fees no later than 30 days from the date of the Publisher's invoice.

3. The Licensee will correspond with the MAPI Research Trust eprovide@mapi-trust.org regarding the availability of translated versions of the Material, if applicable.

4. The Licensee will not make any changes to the Material as supplied by the Publisher or by the MAPI Research Trust, without first consulting the Publisher.

5. The Licensee hereby agrees to delete the Word file containing the Material as soon as the agreed number of administrations have been reproduced.

6. All Material must remain under the management of the Licensee at all times and following use, must be returned to the possession of the Licensee, who is a qualified and registered GL Assessment test user in relation to the scoring and interpretation of the data from the use of the Material. The HADS manual shall be used for scoring and interpretation and is available from the Publisher (see Section 3 above).

7. The Licensee will include the following copyright and acknowledgement notice ("the Copyright Notice") in full on each copy of the Material:

   HADS copyright © R.P. Snaith and A.S. Zigmond, 1983, 1992, 1994.
   Record form items originally published in Acta Psychiatrica Scandinavica 67, 361–70,
   copyright © Munksgaard International
   Publishers Ltd, Copenhagen, 1983.
   This edition first published in 1994 by nferNelson Publishing Company Ltd (now GL Assessment Ltd),1st Floor Vantage London, Great West Road, Brentford TW8 9AG
   GL Assessment Ltd is part of the GL Education Group.
   www.gl-assesssment.co.uk

   This work may not be photocopied or otherwise reproduced by any means, even within the terms of a Photocopying Licence, without the written permission of the Publisher.

8. The Material must not be reproduced in any publication or journal, whether in print or electronic formats, resulting from the research study nor should the Material be used in any way other than that described above.

9. The Licensee will send to the Publisher as soon as possible one copy of any published article, report or publication of the data collection and analysis resulting from the use of the Material. The Publisher does not require details of confidential subject/participant data generated by use of the Material.

10. The Licensee undertakes to and shall procure that all permitted users of the Materials shall exercise the utmost vigilance in protecting the Publisher's copyright privileges on the material involved, both in the English language and as translated. Unauthorised persons must not be given access to these materials and the Copyright Notice must appear in full on each copy of the Material.

11. The Publisher makes no representation or warranties regarding the accuracy or fitness for purpose of the Material. The Publisher shall have no liability for any result, output or determination following the use of the Material or for any conclusions drawn from such use. To the fullest extent permitted by applicable law, the Publisher expressly excludes all warranties either express, implied or statutory in relation to the Material. To the fullest extent permitted by law, the Publishers liability to the Licensee for any costs, expenses, loss or damage (whether

direct or indirect) arising from this Agreement shall not exceed the amount of the TOTAL COPYRIGHT FEE.

12. A person who is not a party to this Agreement has no rights under the Contracts (Rights of Third Parties) Act 1999 to enforce any term of this Agreement but this does not affect any right or remedy of a third party that exists or is available apart from that Act.

13. The Licensee shall not assign or in any way transfer this Agreement without the prior written consent of the Publisher.

14. This Agreement shall be terminated without further notice in any of the following circumstances:

(a) If the Licensee fails to make any payment specified in this Agreement on the due date;

(b) If the Licensee shall at any time be in breach of any of the terms and conditions of this Agreement and if capable of being remedied, such breach is not remedied within 15 days of receipt of written notice thereof; or

(c) If the Licensee is declared insolvent or bankrupt or goes into liquidation (other than voluntary liquidation for the purpose of reconstruction only) or if a Receiver is appointed or if the Licensee is subject to any similar event anywhere in the world.

Termination shall be without prejudice to any monies which may be due to the Publisher from the Licensee and without prejudice to any claim which the Publisher may have for damages and/or otherwise.

Upon termination of this Agreement for any reason the Licensee shall immediately cease to use the Material.

15. Anti-Bribery and Corruption : "the Licensee" shall (i) comply with all applicable laws, statutes, regulations relation to anti-bribery and anti-corruption (Relevant Requirements); (ii) undertake not to engage in any activity, practice or conduct which would constitute an offence under the Relevant Requirements; (iii) have and shall maintain in place throughout the Term of this Agreement its own policies and procedures to ensure compliance with the Relevant Requirements and will enforce them where appropriate. The Parties agree that breach of this section by either party shall be deemed a material breach of this Agreement.

16. Counterparts; Electronic Signatures: Where permitted according to applicable law, this Agreement may be executed in two or more counterparts, each of which shall be deemed an original, and all such counterparts together shall constitute one and the same instrument. Where counterparts are not permitted according to applicable law, this Agreement must be executed (i) either in paper form, in as many original copies as there are parties to the agreement, each copy to be signed in full by each party on the same instrument, or (ii) in electronic form through a validated electronic signing software, where the electronic version is signed in full by each party on the same electronic instrument. Electronically executed or electronically transmitted (including via fax) signatures shall have the full force and effect of original signatures.

17. This Agreement constitutes the entire agreement between the parties in respect of the Material and supersedes all prior oral or written proposals, agreements or undertakings concerning the same.

18. This Agreement shall not be amended or modified in any way other than by an agreement in writing and signed by both parties or their duly authorised representatives and shall come into effect on receipt of the payment in full as specified above and a counter-signed copy of this Agreement.

19. This Agreement shall be governed by and construed in all respects in accordance with English Law and the courts of England and Wales shall have exclusive jurisdiction to settle any dispute

GL Assessment

AS WITNESS THE HANDS OF THE PARTIES
hereto the day and year first above written

Signed for and on behalf of: GL Education Group
GL Education Group Limited
Unit 28 Bramble Road
Swindon
SN2 8HB
United Kingdom
Company number: 02603456
M. A Pritchard 01/09/2020
Rights & Permissions Department

Signed on behalf of GL Assessment Limited

....

Signed by the Licensee: ***Please print this page, sign, and attach this signature page as a scanned document along with your typed User Agreement form, sent as a Word document. Electronic signatures are acceptable.***

User's Signature (handwritten):	**Company/Organisation Stamp (if applicable):**
[signature]	
Title: Associate Lecturer.	
Company/Organisation: University of Sierra Leone.	
Date: 12/08/2020	

PLEASE READ THE FOLLOWING NOTES <u>BEFORE</u> YOU COMPLETE THIS AGREEMENT

Note 1. Licensee's Name	This is the contracting party for the permissions agreement.<br><br>Please ensure the Licensee is authorised to sign legally binding documents on behalf of the contracting organisation.
Note 2. Vat Number	This is only applicable to EU countries. If you do not include your VAT number and are based in the EU, you will be charged VAT at 20% on your invoice.
Note 3. Details of where invoice should be sent.	If the invoice needs to be sent to someone other than the Licensee whose details have been provided on this form already, you must provide the full details of where and to whom the invoice should be sent.<br><br>Please include the FULL name and address, contact telephone number and **email address**.<br><br>If you require a purchase order number, study number or any other specific detail to appear on the invoice in order to have it processed, please include this information in section 5 above.<br><br>The invoice can not be sent elsewhere/changed once it has been created.<br><br>All payments must be made in £ sterling by credit card or cheque drawn on a UK bank or sterling funds transferred directly to the GL Assessment bank account – details of which will be included on the invoice.<br><br>**<u>To make a payment:</u>**

	If you require a purchase order number, study number or any other specific detail to appear on the invoice in order to have it processed, please include this information in section 5 above.<br><br>The invoice can not be sent elsewhere/changed once it has been created.<br><br>All payments must be made in £ sterling by credit card or cheque drawn on a UK bank or sterling funds transferred directly to the GL Assessment bank account – details of which will be included on the invoice.<br><br>**To make a payment:**<br><br>**Proforma invoices**: to pay by credit card, please contact the permissions department on (UK) 0800 6521019 (Int) +44 800 652 1019<br>If you are unable to call to make a payment, please contact permissions@gl-assessment.co.uk for alternative arrangements.<br><br>**Sales invoices:** please contact credit control on 01793 516347 int +44 1793 516347<br>If you are unable to call to make a payment, please contact permissions@gl-assessment.co.uk or credit.control@gl-assessment.co.uk for alternative arrangements.
Note 4.<br>GL Reader Code<br>GL Qualification Code	GL Assessment products are restricted to qualified and registered users.<br><br>You will be issued with a Reader Code and Qualification Number once your application has been processed.<br><br>If the Licensee for the study is different to that of the registered user, please provide the name of the registered user along with the reader code and qualification number in section 4 above.
Note 5.<br>University Course and Supervisor's Name/Supervisor's GL Reader Code. / qualification code	Complete only if you are an undergraduate student and not qualified to register with GL Assessment yourself. Your supervisor must register and sign an agreement on your behalf.
Note 6. Details of project	Ensure you include study title, project title, name of study group.
Note 7. Total number of Administrations	Administrations mean the number of times the scale is to be used not the number of participants/subjects in the study i.e. test to be administered 3 times to 50 participants/subjects = 150 administrations.<br><br>If the study is international it should include the total number of administrations, whatever number of countries/languages involved (**excluding** those administered in the German language; see below).

Note 8. Translations of the HADS/GHQ	The HADS questionnaire is distributed in its translated forms by the MAPI Research Trust.<br><br>**For all queries re. availability and status of translations, please contact Mapi Research Trust in France at** eprovide@mapi-trust.org Tel: +33 472 13 65 75<br><br>Please note that further costs, in addition to those charged by GL Assessment for the use of the scale(s), may be charged by the MAPI Research Trust when obtaining the translations of the scale. You must liaise directly with the MAPI Research Trust regarding translations and any additional associated fees.<br><br>Each new translation must undergo a full linguistic validation process by Mapi Research Institute, according to standard recognized methodology of translation, as described in Acquadro C, Conway K; Giroudet C, Mear I. Linguistic Validation Manual for Patient-Reported Outcomes (PRO) Instruments. Mapi Research Institute, 2004.<br><br>**Please note that GL Assessment do not hold the rights to the GERMAN translation of the HADS, therefore you will be unable to obtain the German translation of the scale either through ourselves or the MAPI Research Trust. If you require the German translation of the HADS you should contact Sylvia.Schlutius@hogrefe.ch at Hogrefe AG, Bern, Switzerland. Please do not include any number of administrations that are intended for German usage on this form as you may be charged twice.**

Ends.
